# Supplementary material for: Genome-Wide Identification of Long Non-coding RNA in Trifoliate Orange (Poncirus trifoliata (L.) Raf) Leaves in Response to Boron Deficiency
Source: Int J Mol Sci. 2019 Oct 31;20(21):5419. doi: 10.3390/ijms20215419 (PMC6862649; doi:10.3390/ijms20215419)
Supplement: Supplementary file 1 [file ijms-20-05419-s001.zip › Supplementary files/Supplementary data captions.rtf]

Supplementary data captions
Supplementary data 1-Table S1. The differential expressed genes under boron deficiency conditions.
Supplementary data 2-Table S2. The differential expressed lncRNAs under boron deficiency conditions.
Supplementary data 3-Table S3. Significantly differentially expressed target genes.
Supplementary data 4-Table S4. LncRNA-mRNA interaction network analysis.
Supplementary data 5-Table S5. The description of all genes of clementine mandarin (Citrus clementina).
Supplementary data 6-Table S6. GO enrichment analysis on target genes related to plant hormone.
Supplementary data 7-Table S7. Primers used for qRT-PCR analysis of lncRNA and mRNA levels.
Supplementary data 8-Figure S1. Cluster analysis of differentially expressed mRNAs (DEMs) and lncRNAs (DELs) in the leaf of trifoliate orange seedling under boron (B) deficient conditions. Hierarchical clustering analysis indicate 115 DELs (A) and 1547 DEMs (B) that were differentially expressed between B deficiency (BD) and control plants (CK). Respectively, the red and the green shades represent the expression levels above and below the relative expression among all samples.
Supplementary data 9-Figure S2. Prediction of lncRNA-mRNA association network. The co-expression network was composed of 558 network nodes and 838 connections between 90 lncRNA and 468 coding genes. This co-expression network indicated that one lncRNA could target 42 coding genes at most and that one coding gene could correlate with 9 lncRNA at most.
