# Supplementary figures and images for: Genome-Wide Identification of Long Non-coding RNA in Trifoliate Orange (Poncirus trifoliata (L.) Raf) Leaves in Response to Boron Deficiency
Source: Int J Mol Sci. 2019 Oct 31;20(21):5419. doi: 10.3390/ijms20215419 (PMC6862649; doi:10.3390/ijms20215419)

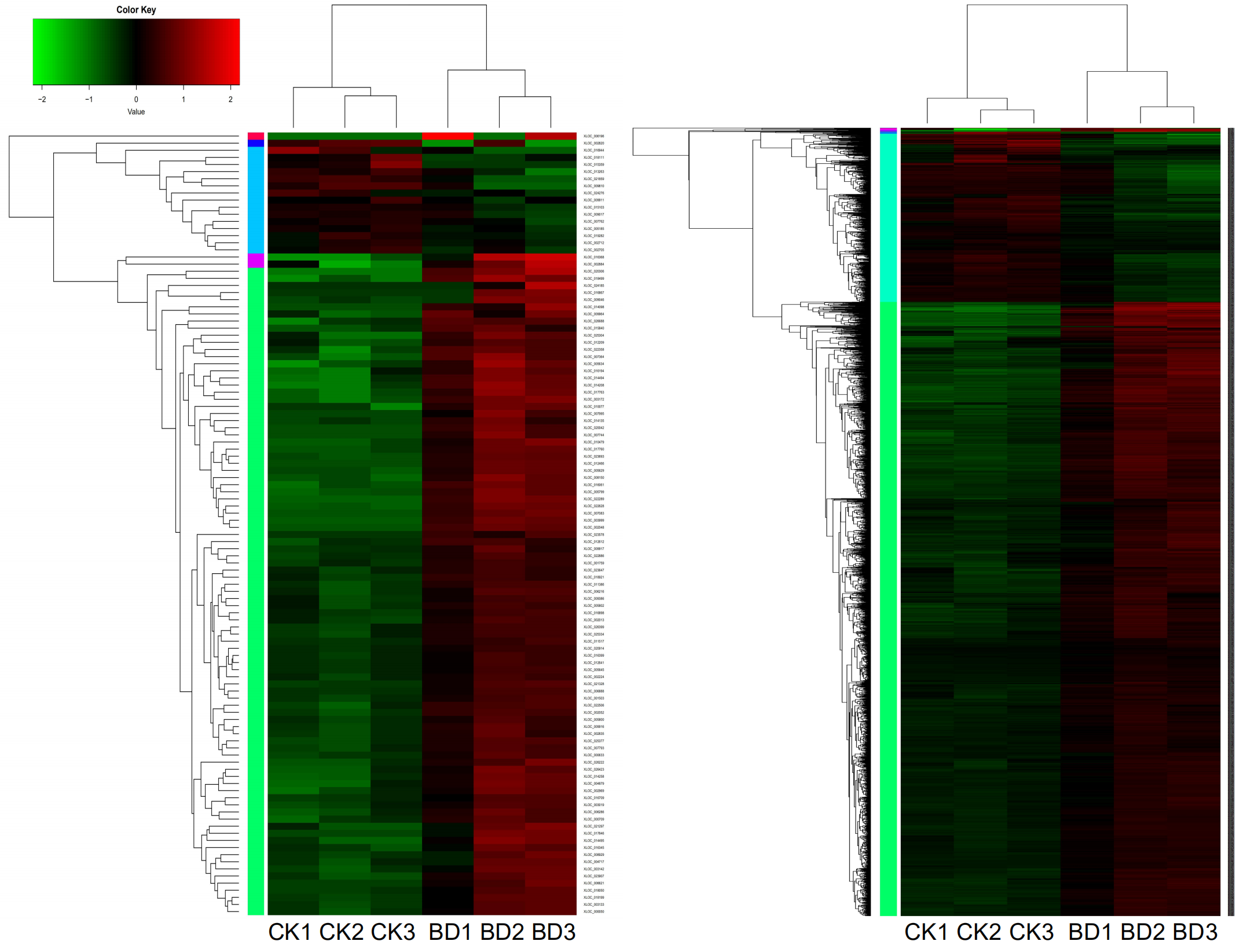

Supplement: Supplementary file 1 [file ijms-20-05419-s001.zip › Supplementary files/Supplementary data 8-Figure S1.tif]

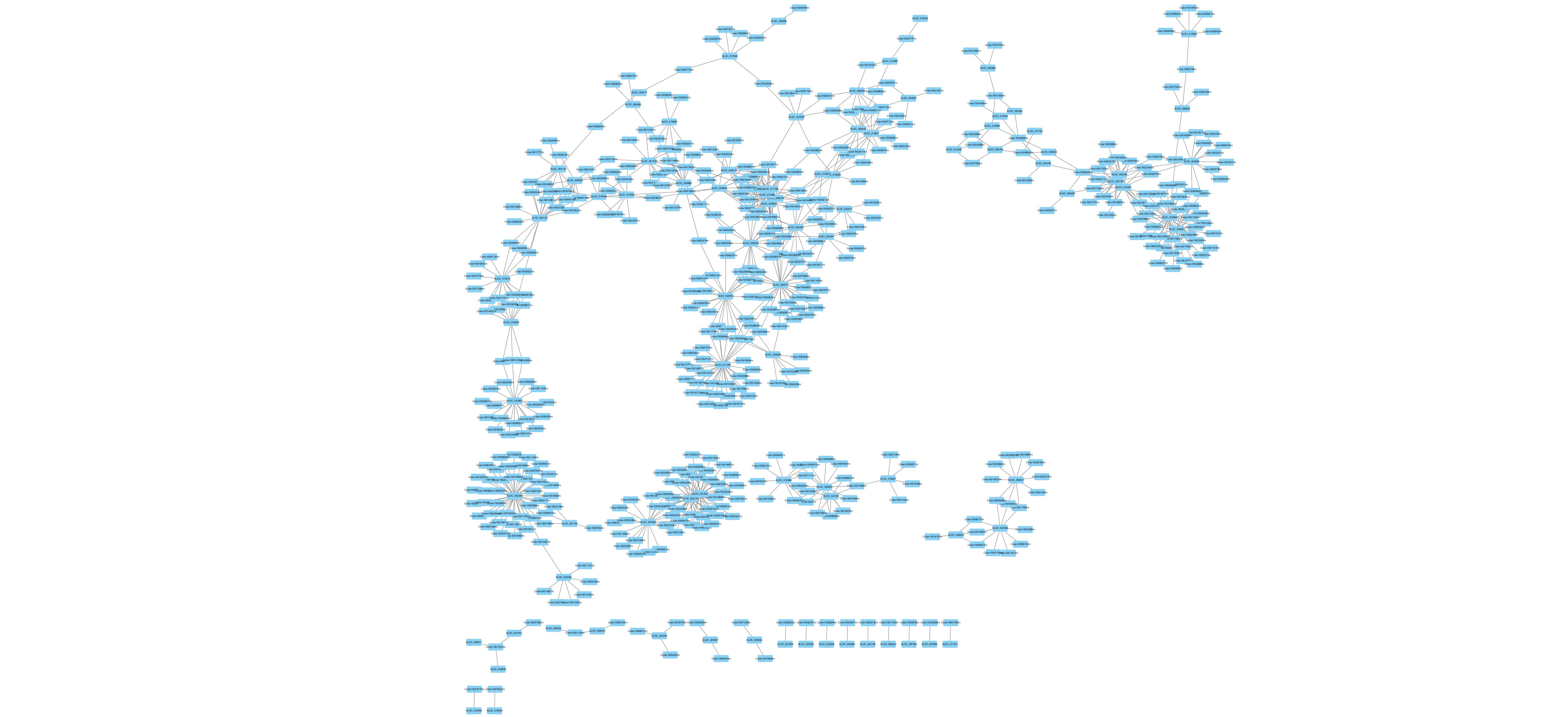

Supplement: Supplementary file 1 [file ijms-20-05419-s001.zip › Supplementary files/Supplementary data 9-Figure S2.jpeg]
